# Accessory Maxillary Ostia: Anatomical and Clinical Relevance From a Cadaveric Case Report

**DOI:** 10.7759/cureus.86882

**Published:** 2025-06-27

**Authors:** Shreya Rangarajan, Wessam Ibrahim

**Affiliations:** 1 School of Medicine, Carle Illinois College of Medicine, University of Illinois Urbana-Champaign, Urbana, USA; 2 Biomedical and Translational Sciences, Carle Illinois College of Medicine, University of Illinois Urbana-Champaign, Urbana, USA

**Keywords:** accessory maxillary ostium, anatomical variation, anatomy, cadaveric dissection, chronic sinusitis, head and neck surgery, maxillary sinus, maxillofacial radiology, nasal cavity, otolaryngology

## Abstract

Accessory maxillary ostia (AMOs) are relatively common anatomical variants of the paranasal sinuses. They present as small, oval or circular openings found at or near the maxillary sinus, in the posterior nasal fontanelle, anterior nasal fontanelle, or, rarely, in the hiatus semilunaris. We present a unique cadaveric report of bilateral AMOs, exploring the causes, associated findings, and anatomical variations within the maxillary sinus.

During the routine dissection of the nasal cavity, three AMOs were discovered in an 84-year-old female donor, who passed away from a myocardial infarction. No other past medical history was obtained from the file due to the anonymous nature of the donation program. On the left side, there was one AMO in the posterior fontanelle, lying parallel to the semilunar hiatus and measuring 7 mm × 4 mm. Upon removal of the middle turbinate, another AMO, lying perpendicular to the semilunar hiatus, was identified, measuring 5 mm × 3 mm. On the right side, there was one AMO in the posterior fontanelle, lying parallel to the semilunar hiatus and measuring 7 mm × 4 mm. When dissecting down to the maxillary sinus, a soft, non-friable, nodular growth was found in the left maxillary sinus, while the right maxillary sinus appeared normal. The primary maxillary ostium in the semilunar hiatus seemed to be normal on both sides, as well as the rest of the nasal cavity.

This case illustrates a rare presentation of AMO, emphasizing the importance of understanding anatomical variants within the paranasal sinuses. The dimensions and positions of AMOs documented in this case report can serve as a valuable reference, helping endoscopic sinus surgeons identify these variants and develop novel techniques to repair them seamlessly. Furthermore, this case presents a unique finding within the maxillary sinus that could explain the presence of multiple AMOs on one side, compared to the other. While the relationship between AMOs and chronic maxillary sinusitis has been well documented, future studies could explore the relationship between AMOs and nodular growths within the maxillary sinus.

## Introduction

The maxillary sinuses are the largest paranasal sinuses, which communicate with the nasal cavity and reside within the maxillofacial region and the skull. They are the first to develop, typically around 17 weeks of gestation, and achieve their final form and structure by the second to third decade of life [1]. Literature indicates that males, on average, have a larger maxillary sinus than females; however, this difference is not statistically significant [1].

One anatomical variant of the maxillary sinus is an accessory maxillary ostium (AMO). These are small, oval or circular openings usually found at or near the maxillary sinus, in the posterior nasal fontanelle, anterior nasal fontanelle, or, rarely, in the hiatus semilunaris [2,3]. They can present unilaterally or bilaterally, most commonly in patients with chronic maxillary sinusitis. AMO is clinically significant, as it may contribute to the persistence of chronic sinusitis by promoting mucus recirculation and impairing mucociliary clearance within the maxillary sinus [1-3]. There has been some discrepancy in the literature regarding whether AMOs are congenital or acquired; however, the prevalence is about 10%-20% of the population, depending on the study, and up to 40% in patients with chronic maxillary sinusitis [4]. 

This case report aims to add to the growing literature about the presentation of AMOs and the clinical implications of this anatomical anomaly. To date, there have been limited cadaveric case reports and studies related to AMOs. Here, we present a unique case of bilateral AMOs found during a cadaveric dissection and explore the causes and associated findings within the nasal cavity as a result of this anatomical deviation. 

## Case presentation

Methods

Following a bilateral standard anatomical dissection technique for the head and neck region - with a focus on the maxillofacial region, the maxillary sinus, and its anatomical relationships within the nasal cavity - the nasal mucosa was carefully reflected to expose the lateral wall of the nasal cavity. The findings were photographed using a digital camera, and relevant measurements were recorded. Descriptive analysis was used to characterize the anatomical features observed. No statistical analysis was performed due to the descriptive nature of the case report. The tissue sample from an identified lesion was sent to the Veterinary Diagnostic Laboratory (VDL) for routine hematoxylin and eosin (H&E) staining.

Results

**Figure 1:**
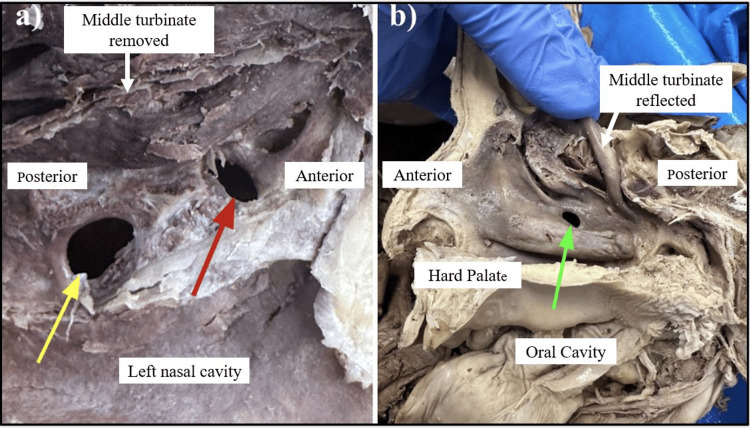
Bilateral accessory maxillary ostium (AMO) (a) Left nasal cavity showing two AMOs: the yellow arrow indicates the AMO in the posterior fontanelle, measuring 7 mm × 4 mm, and the red arrow indicates the AMO in the semilunar hiatus, measuring 5 mm × 3 mm, after the complete excision of the middle turbinate. (b) Right nasal cavity showing a large AMO (green arrow, measuring 7 mm × 4 mm) in the middle meatus after reflection of the middle turbinate.

Three AMOs were identified in an 84-year-old female donor who passed away from a myocardial infarction. No other past medical history was obtained from the file due to the anonymous nature of the donation program. On the left side, one AMO in the posterior fontanelle, lying parallel to the semilunar hiatus, measured 7 mm × 4 mm, as indicated by the yellow arrow in Figure 1.

**Figure 2:**
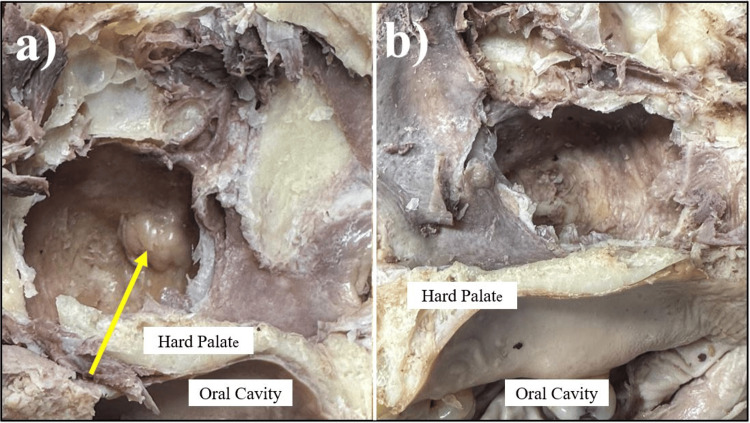
Nodular mass in the left maxillary sinus (a) Left maxillary sinus opened, showing a nodular mass growth (yellow arrow). (b) Right maxillary sinus opened, with no abnormal findings noted.

Upon removal of the middle turbinate, another AMO, lying perpendicular within the semilunar hiatus, was identified, measuring 5 mm × 3 mm (Figure 1). On the right side, one AMO in the posterior fontanelle, lying parallel to the semilunar hiatus, measured 7 mm × 4 mm. When dissected down to the maxillary sinus, a soft, non-friable, nodular growth was found in the left maxillary sinus. In contrast, the right maxillary sinus appeared normal (Figure 2).

**Figure 3:**
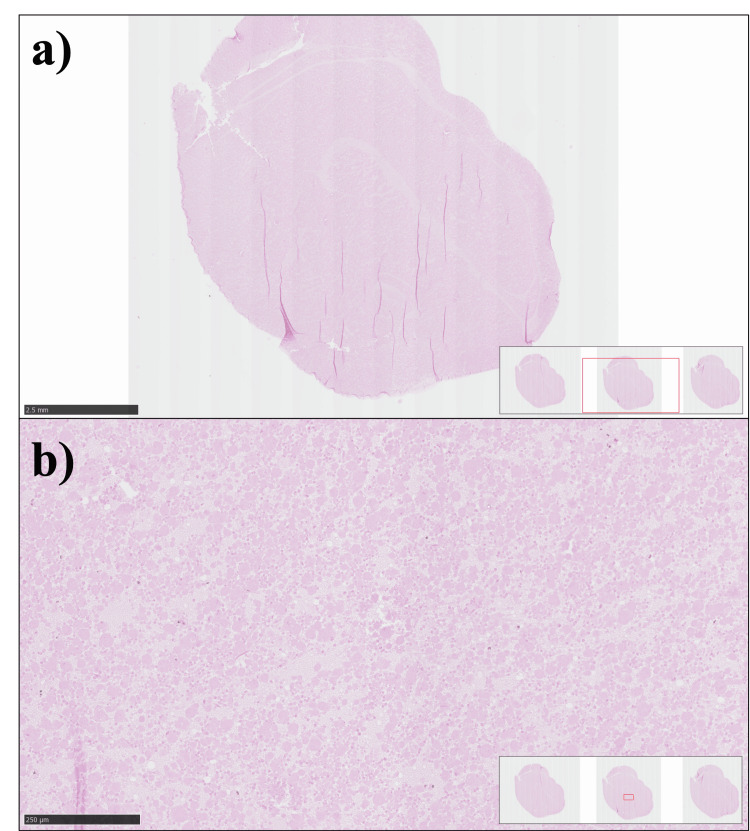
Ruptured mucus retention cyst (a) Hematoxylin and eosin (H&E)-stained section showing a ruptured mucous retention cyst at low magnification (scale bar: 5 mm). The section shows a large cystic cavity/space with characteristic features of a mucous retention cyst that has undergone rupture, with disrupted architecture, and no true epithelial wall present. (b) Higher magnification H&E-stained section of the ruptured mucous retention cyst (scale bar: 250 μm), showing evidence of chronic inflammatory infiltrate composed of epithelioid macrophages, lymphocytes, and other inflammatory cells.

A nodular mass growth, later identified as a mucous retention cyst, was collected and sent to the pathology lab for processing with H&E staining, the results of which are shown in Figure 3. The histology of the ruptured cyst shows infiltration with epithelioid macrophages and chronic inflammatory infiltrates. The primary maxillary ostium in the semilunar hiatus appeared normal on both sides, and the rest of the nasal cavity appeared normal as well.

## Discussion

AMOs are common anatomic variants in the paranasal sinuses and are often found incidentally on imaging or nasal endoscopy. Here, we present a case of three AMOs that were found incidentally during a routine anatomical dissection of the nasal cavity. In this case, the size of the AMOs, and the rare presentation of an AMO within the semilunar hiatus, warrant further discussion.

The largest AMO in the literature to date was measured to be 10 mm; however, the average size of an AMO is usually between 0.5 mm and 5 mm [5]. The relationship between the size of an AMO and the severity of conditions such as chronic maxillary sinusitis remains unclear. However, there is a relationship between the presence of an AMO and chronic maxillary sinusitis. Bani-Ata et al. found a strong association between the presence of an AMO and chronic sinusitis [4]. Similarly, Swain’s review article found that the presence of an AMO was associated with a two-fold increase in chronic maxillary sinusitis. In addition, the presence of an AMO indicated a higher likelihood of a patient also having a mucous retention cyst within the maxillary sinus, as well as mucosal thickening [6,7]. This link appears to be mediated by impaired mucociliary clearance due to mucus recirculation between the natural and accessory ostia, perpetuating inflammation within the sinus cavity.

The location of the AMO deserves further exploration. AMOs commonly arise in the posterior fontanelle of the middle meatus. One likely explanation for this finding is related to how chronic maxillary sinusitis obstructs the primary maxillary ostium in patients, leading to the formation of an AMO to relieve the blockage [8]. The presence of an AMO in the semilunar hiatus is relatively uncommon, in comparison.

To date, a significant area of debate regarding AMOs centers on whether they are congenital or acquired. Orhan Soylemez and Atalay examined the presence of AMOs via computed tomography (CT) scans in children <13 years old and patients >13 years old [9]. They found that the presence of AMOs in the <13-year-old age group was lower compared to the AMOs found in patients aged 13 years or older. Their study showed that AMOs were more likely to develop after the sinuses had fully developed, indicating that AMOs are likely acquired rather than congenital. 

Recent studies published within the last five years provide further details regarding the anatomic location of an AMO and potential clinical associations. An extensive retrospective study by Serindere et al., involving 400 patients, demonstrated significant associations between the presence of AMOs and multiple sinonasal variants. The study had an AMO prevalence of 10.5%, and patients with AMOs were also likely to have an increased incidence of agger nasi cells, Haller cells, nasal septum deviation, and hypertrophy of the inferior concha. Of note, an AMO was associated with a 39% increase in maxillary sinusitis (OR = 1.39) and a decreased incidence of mucus retention cysts (OR = 0.50). No significant association with mucosal thickening was observed in this study [10].

Complementing the above findings, Do and Han in 2022 employed three-dimensional (3D) CT analysis to characterize the spatial dimensions and anatomical relationships of AMOs. In their cohort of 197 patients, the prevalence of AMOs was 21.3%, and most were located 5.4 mm posterior and 0.7 mm inferior to the natural ostium. The mean size of the AMOs was 2.8 mm horizontally and 3.0 mm vertically. Interestingly, this study reported a statistically significant association between AMO presence and mucosal thickening (p = 0.029), which differs from the study by Serindere et al. [10]. The measurements reported in this study emphasize that, although AMOs are often small, they may still significantly affect sinus physiology [11].

Our findings indicate the significant role of the AMO in sinus dynamics, as their presence can disrupt normal mucociliary clearance and promote mucus recirculation between the natural and accessory ostia, which may contribute to the persistence or recurrence of chronic maxillary sinusitis [7,11]. In surgical contexts, awareness of the anatomical location of the AMO is crucial for endoscopic sinus surgeons, as these additional openings can be utilized for irrigation or as alternative drainage routes during functional endoscopic sinus surgery [7,12]. Using high-resolution CT scans or direct visualization with diagnostic nasal endoscopy is essential in diagnosing AMOs in patients [11]. These imaging modalities enable precise localization and assessment of AMOs, as well as their relationship to surrounding structures, thereby supporting both accurate diagnosis and effective surgical planning.

In our case, the locations and sizes of AMOs contribute to understanding the spectrum of anatomical presentations, emphasizing the need for a thorough exploration in both clinical and research settings.

## Conclusions

This case presentation adds to the growing body of literature on AMOs, particularly in the context of cadaveric dissection. Studies to date have retrospectively examined the origin of AMOs and the relationship between AMOs and other nasal pathologies, such as chronic maxillary sinusitis and nasal polyps. Future studies could investigate the prevalence of multiple AMOs in patients and consider how the size and shape of the AMOs impact the severity of chronic maxillary sinusitis. Furthermore, applying high-resolution imaging may enhance understanding of these clinically relevant anatomical variants.
